# Herbal medicines in the treatment of tinnitus: An updated review

**DOI:** 10.3389/fphar.2022.1037528

**Published:** 2023-01-04

**Authors:** Dongliang Liu, Yue Hu, Dali Wang, Hezhou Han, Yi Wang, Xilu Wang, Zhaoyu Zhou, Xiulan Ma, Yaodong Dong

**Affiliations:** Department of Otolaryngology Head and Neck Surgery, Shengjing Hospital of China Medical University, Shengyang, China

**Keywords:** tinnitus, clinical trials, herbal medicine, medicinal plants, dementia, depression, mood disorders

## Abstract

Tinnitus is perception of sound in the absence of an apparent external acoustic stimulus. The condition is prevalent in adults, especially the elderly (≥65 years), and may be associated with cognitive function decline and significantly impacts on the quality of life, heralding difficulties in managing this challenging disorder. Interventions for tinnitus have been varied. However, drugs have not yet been approved for the treatment of tinnitus and there is no pharmacotherapy recommended by existing guidelines. Still, herbal medicines are used for the treatment of tinnitus in many countries, especially *Gingko* (G.) *biloba.* In the current updated literature review, we evaluated the efficacy of herbal medicines in the treatment of tinnitus by reviewing the evidence of relevant randomized controlled trials. The authors also highlight some of the issues in clinical trials of herbal medicines given that currently available evidence on herbal medicines for tinnitus is overall of insufficient quality and the conclusions from existing trials are conflicting. Nevertheless, there is a clear and urgent need for safe and effective pharmacotherapy of tinnitus.

## Introduction

Tinnitus is prevalent in adults, especially the elderly (≥65 years), and can be categorized to subjective tinnitus and objective tinnitus (Baguley et al., 2013; Han et al., 2021). Subjective tinnitus is defined as conscious awareness of sound in the absence of an apparent external acoustic stimulus which can only be perceived by the affected person while objective tinnitus can also be perceived by the examiner. Tinnitus can be primary without an apparent cause or secondary where a specific cause can be identified. Tinnitus affects approximately one in 10 adults in the United States (Bhatt et al., 2016). In United Kingdom, about 15% of the adult population suffer from the disorder (Biswas and Hall, 2021). According to a cross-sectional analysis of data in the United Kingdom Biobank (Dawes et al., 2020), approximately six percent of 168,348 participants aged between 40 and 69 years with hearing difficulties and tinnitus reported annoying tinnitus. The prevalence of tinnitus varies widely, from 4.3% to 51.3%, in China (Zhang et al., 2021). The presence of chronic tinnitus may be associated with cognitive function decline, especially decline of attention and emotional health, anxiety and depression (Trevis et al., 2018).

In addition, tinnitus can significantly impact on the quality of life of individuals (National Guideline, 2020) and incur an increasing economic cost (Stockdale et al., 2017). The direct and indirect costs of tinnitus treatment are considerable, and there is a direct relationship between tinnitus severity and associated costs (Trochidis et al., 2021). Aging, unhealthy lifestyles, systemic diseases, sleep disorder, exposure to noise, depression and various anxiety disorders can induce or exacerbate tinnitus (Veile et al., 2018; Chamouton and Nakamura, 2021; Dai et al., 2021; Schubert et al., 2021), heralding difficulties in managing this challenging disorder.

There are many interventions for tinnitus, including educational counselling, relaxation therapy, tinnitus retraining therapy, cognitive behavioral therapy, sound therapy, transcranial direct current stimulation, repetitive transcranial magnetic stimulation, transcutaneous electrical nerve stimulation, acupuncture, and pharmacotherapies (Langguth et al., 2019). Hitherto, no drug has been approved for the treatment of tinnitus by regulatory agencies around the world. Due to the lack of effective treatment for tinnitus, it is a common practice in many countries that dietary supplements are used for treatment of tinnitus, especially *Gingko* (G.) *biloba* and lipoflavones (Bhatt et al., 2016; Coelho et al., 2016) although these alternative treatments are not endorsed by regulatory bodies such as the American Academy of Otolaryngology-Head and Neck Surgery (AAO-HNSF) and the European guidelines. According to alternative medicine theories, dietary therapy is worthwhile considering its potential benefits, good tolerabilities and cultural acceptability (Luetzenberg et al., 2020). Enrico et al. (2007) argue that the use of complementary and alternative medicine products in the treatment of tinnitus often lacks substantial scientific support and that these substances may not be clinically effective either. No definitive conclusions can be drawn regarding the pharmacological approach to complementary and alternative medicine in the treatment of tinnitus. Hofmeister and Coelho also pointed out that further high-quality analytical studies should be conducted before recommendation by clinicians (Coelho et al., 2016; Hofmeister, 2019).

**FIGURE 1:**
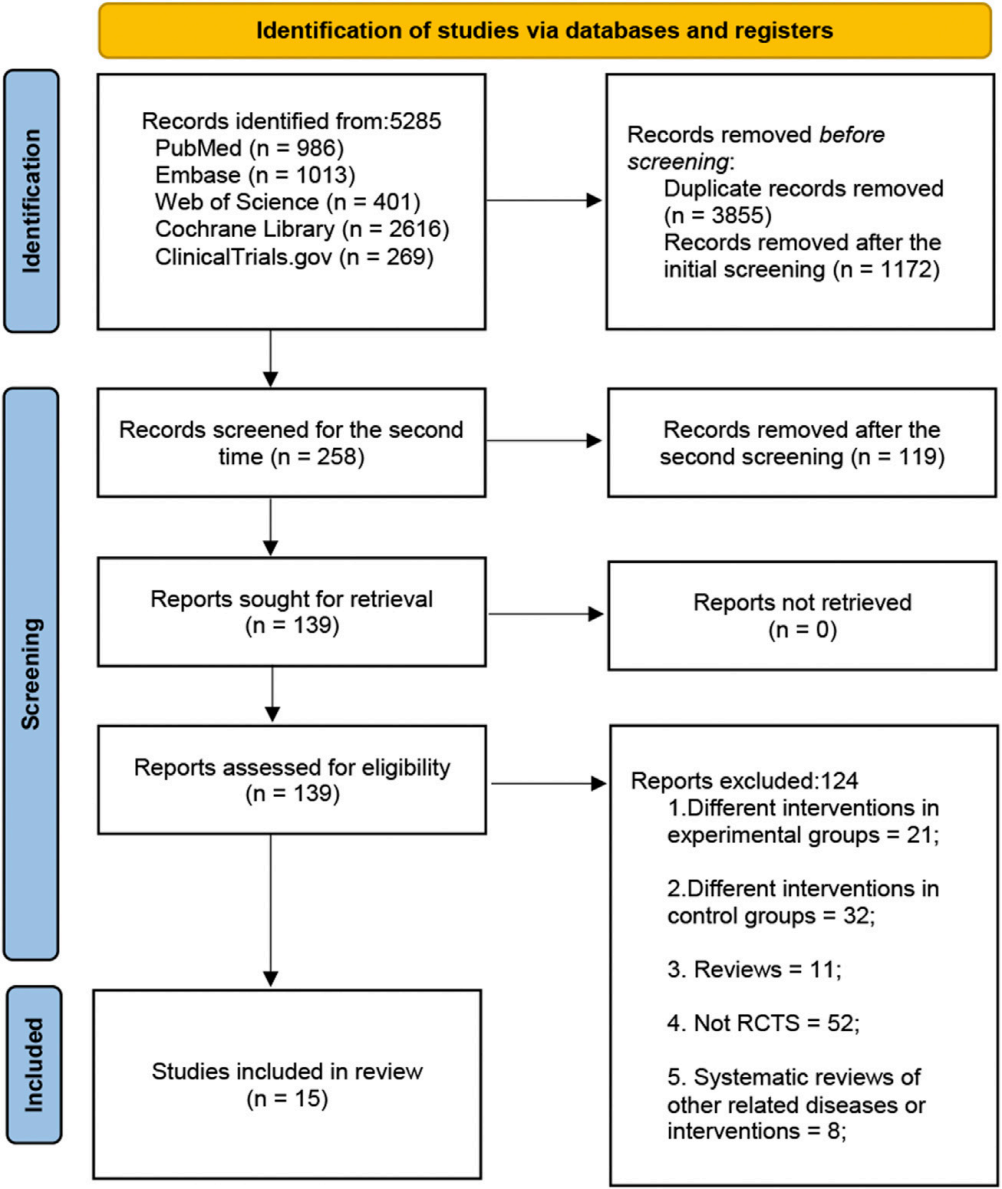
PRISMA flowchart for study.

We are interested in whether herbal medicines are safe and effective in the treatment of tinnitus. In the current updated literature review, the authors evaluated the efficacy and safety of herbal medicines in the treatment of tinnitus by reviewing the evidence of relevant randomized controlled trials (RCTs). The authors searched PubMed, Embase, Web of Science, Cochrane Library and ClinicalTrials.gov for original studies on tinnitus from the date of inception to 1 February 2022. Literature with the keyword “tinnitus” in the title/abstract was eligible, with filters for “randomized controlled trial” and “controlled clinical trial.” Randomized placebo-controlled studies or studies with an active comparator were eligible. There was no language restriction. In addition, manual search was performed for additional eligible articles from the reference lists of review articles, clinical guidelines, and pairwise meta-analyses. Measures of tinnitus included Tinnitus Handicap Inventory (THI) score (Newman et al., 1996), tinnitus loudness, Visual Analogue Scale (VAS) score, Tinnitus Functional Index (TFI), Tinnitus Questionnaire (TQ) score (Richard, 1996) and effectiveness. Totally 5,285 records were identified, and 15 studies were eligible. The search process is illustrated in Figure 1. Eligible RCTs on herbal medicines in the treatment of tinnitus are summarized in Supplementary Table S1.

### *G. biloba* in the treatment of tinnitus

Ginkgo [Ginkgoaceae; Gingko; *G. biloba* L*.*, synonym, *Salisburia biloba*, *Salisburia adiantifolia*] has been used as a medicinal herb for over two thousand years. The leaves of *G. biloba* have been used for the treatment of central nervous system illnesses (Belwal et al., 2019), including Alzheimer’s disease (Nowak et al., 2021), metabolic syndrome (Eisvand et al., 2020), cardiovascular diseases (Tian et al., 2017) and a variety of other conditions (Roland and Nergård, 2012). *G. biloba* has been shown to protect against oxidative stress, inhibit platelet-activating factor (PAF), suppress inflammation, affect vascular smooth muscle, inhibit amyloid aggregation, and modulate gene expression (DeKosky et al., 2008). *G. biloba* has an abundance of bioactive compounds, and the main constituents of *G. biloba* leaf extract include bioflavonoids and flavonoids (such as quercetin, kaempferol, and isorhamnetine), terpene trilactones (such as ginkgolides and bilobalide), polyprenols, and organic acids (Guo et al., 2020). Standard *G. biloba* extract, including *G. biloba* extract EGb 761, (Rökan, Tanakan, Tebonin), contains approximately 24% flavone glycosides and 6% terpene lactones (2.8%–3.4% ginkgolides A, B, and C and 2.6%–3.2% bilobalide) (EGb, 2003; Unger, 2013). Dietary flavonoids are bioactive compounds that have been extensively studied for their relationship to vascular health outcomes. Because tinnitus development is associated with vascular access, dietary flavonoids such as those present in *G. biloba* extract have antioxidant and vasodilatory effects and may play a role in alleviating tinnitus symptoms. Preclinical and clinical studies have shown that apart from its antioxidant and vasodilatory effects, *G. biloba* extract may exert its actions by improving cochlear microcirculation, protects against ototoxicities, and alleviates aging associated degeneration (Barth et al., 2021).

*G. biloba* studies in the treatment of tinnitus are listed in Table 1. Though *G. biloba* extract has been investigated for the treatment of tinnitus in a number of clinical studies, including RCTs, its efficacy remains inconclusive or questionable according to recent meta-analyses (Mahmoudian-Sani et al., 2017; Kramer and Ortigoza, 2018; Spiegel et al., 2018; Sereda et al., 2022). An early randomized study of 259 patients with tinnitus of less than 1 year duration showed that *G. biloba* extract (Meyer, 1986) reduced the severity of tinnitus in 70% of the patients. A double-blind placebo-controlled trial of 1,121 healthy subjects with tinnitus showed that 50 mg *G biloba* extract LI 1370 (containing 25% flavonoids, 3% ginkgolides, and 5% bilobalides) three times daily for 12 weeks resulted in no notable improvement in tinnitus versus placebo (Drew and Davies, 2001; Rejali et al., 2004; Polanski et al., 2016). The authors also failed to find improvement in other symptoms of cerebral insufficiency with *G. biloba* extract. Overall, *G. biloba* extract was safe and had no serious side effects. In a randomized placebo-controlled double-blind trial, tinnitus patients received *G. biloba* extract 120 mg/day or placebo for 12 weeks. No significant difference in changes in THI scores was observed between subjects receiving *G. biloba* extract and those receiving placebo (*p* = .51) (Rejali et al., 2004). In a crossover RCT, patients with tinnitus received clonazepam (.5 mg per tablet) or *G. biloba* (40 mg per tablet) for the first 3 weeks and switched to the other drug after a 2-week washout and for the final 3 weeks, subjects were instructed to increase the dose by 40 mg every 3 days to a maximum of 160 mg daily until they perceived a satisfactory decrease in tinnitus loudness or intolerable side effects. The study found that *G. biloba* had no significant effect on tinnitus loudness, duration, annoyance, and THI score (Han et al., 2012). In an open phase study followed by a double-blind placebo-controlled study, 80 patients with persistent severe tinnitus received *G. biloba* (15 mg bd) in the open phase and 20 of 21 patients who reported a positive effect in the open phase went on to receive *G. biloba* or placebo. No significant effect on tinnitus was observed (Holgers et al., 1994).

**TABLE 1:** *G. biloba* in the treatment of tinnitus.

	Study design (trial registration)	Patients	Interventions	Outcomes
*G. biloba* extract EGb 761 Morgenstern and Biermann, (2002)	RCT	Chronic tinnitus aurium (*n* = 60)	Patients received*G. biloba* extract EGb 761 2 × 80 mg/d subsequent to 10-day EGb 761 infusion treatment (35 patients) or placebo (38 patients)	Significant change in tinnitus volume with *G. biloba* extract EGb 761
*G. biloba* extract Rejali et al. (2004)	A randomized placebo-controlled double-blind trial	Tinnitus (n = 66)	Patients received*G. biloba* extract 120 mg once daily sustained release formulation or placebo for 12 weeks	A-4.7 ± 12.1 reduction in THI score vs. placebo *-*2.2 ± 16.7, *p* = .51
*G. biloba* extract Han et al. (2012)	An open-label, randomised, crossover study	Tinnitus (*n* = 38)	Patients received clonazepam or *G. biloba* for the first 3 weeks. For the next 2 weeks of washout no medication was taken. For the final 3 weeks, subjects were given the other drug. The initial dose of clonazepam and *G. biloba* was one tablet daily (clonazepam .5 mg; G biloba 40 mg). Subjects were instructed to increase the dose by one tablet every 3 days to a maximum of four tablets daily until they perceived a satisfactory decrease in tinnitus loudness or intolerable side effects	*G. biloba* had no significant effect on tinnitus loudness, duration, annoyance, and tinnitus handicap inventory score
*G. biloba* Holgers et al. (1994)	An open phase study followed by a double-blind placebo-controlled study	Persistent severe tinnitus (*n* = 80)	Patients received *G. biloba* (15 mg bd) in the open phase (80 patients) and 20 of 21 who reported a positive effect went on to receive *G. biloba* (7 patients), placebo (7 patients), or either (6 patients)	No significant effect on tinnitus
*G. biloba*extract EGb 761 Procházková et al. (2018)	Double blind RCT	Sub-chronic or chronic tinnitus (*n* = 200)	Patients received 120 mg *G biloba*extract EGb 761 or 600 mg pentoxifylline, each twice a day for 12 weeks	*G. biloba* led to a significant least square mean (LSM) reduction from baseline in the abridged TQ score (−1.57, 95%CI − 2.25 to − .89; *p* < .001), the 11-Point Box Scale for tinnitus loudness (−.41, 95% CI − .68 to − .15, *p* = .002) and annoyance (−.56, 95% CI− .84 to − .27; *p* < .001)
*G. biloba* dry extract Polanski et al. (2016)	Double-blind RCT	Tinnitus with sensorineural hearing loss (*n* = 58)	Patients received*G. biloba* dry extract (120 mg/day), α-lipoic acid (60 mg/day) + vitamin C (600 mg/day), papaverine hydrochloride (100 mg/day) + vitamin E (400 mg/day), and placebo	No difference in THI before and after treatment with *G. biloba* dry extract

*G. biloba* extract EGb 761 is the most widely tested drug in both non-clinical tinnitus models as well as in clinical trials. The extract is adjusted to 22.0%–27.0% *G.* flavonoids calculated as ginkgo flavonole glycosides and 5.0%–7.0% terpene lactones which consist of 2.8%–3.4% ginkgolides A, B, C and 2.6%–3.2% bilobalides and contain less than 5 ppm ginkgolic acids (Barth et al., 2021). A recent double-blind RCT of subjects with sub-chronic or chronic tinnitus showed that *G. biloba* extract EGb 761,120 mg, twice daily for 12 weeks caused a significant reduction from baseline in the least square mean (LSM) of the abridged TQ score (LSM − 1.57, 95%CI − 2.25 to − .89; *p* < .001), the 11-Point Box Scale for tinnitus loudness (LSM − .41, 95% CI − .68 to − .15, *p* = .002) and annoyance (LSM − .56, 95% CI− .84 to − .27; *p* < .001). The proportion of subjects with an abnormal Hospital Anxiety and Depression Scale (HADS) score decreased from 36% at baseline to 23% post treatment with *G. biloba* extract EGb 761 (*p* = .005). Furthermore, *G. biloba* extract EGb 761 had a lower rate of adverse events than its comparator pentoxifylline. This finding was supported by another parallel group RCT showing that 240 mg *G biloba* extract EGb 761 significantly improved self-perception of tinnitus loudness and severity after 90 days of treatment in patients with hearing loss (Procházková et al., 2018; Nishad et al., 2019; Radunz et al., 2020). In an earlier RCT of 60 patients with chronic tinnitus, Morgenstern and Biermann demonstrated that 160 mg/day *G biloba* extract EGb 761 subsequent to 10-day *G. biloba* extract EGb 761 infusion resulted in significant change in tinnitus volume compared to placebo (Morgenstern and Biermann, 2002). In a systemic review of eight clinical studies involving 1,199 patients, Boetticher found that *G. biloba* extract EGb 761 in sufficient dosing and treatment duration was effective for tinnitus compared to placebo (von Boetticher, 2011). The author attributed the failure of *G. biloba* extracts in reducing severity of tinnitus in some trials to the types of *Ginkgo biloba* extracts used or flaws in the trials. The inconclusive results of *G. biloba* extracts for tinnitus have led some investigators to question whether *G. biloba* extracts should be used for treatment of tinnitus (Smith et al., 2005). The inconsistencies in trial results of *G. biloba* extract highlight the need for further delineation of *G. biloba* extract components, standardization of *G. biloba* extract contents, and optimization of *G. biloba* extract doses and treatment duration in future clinical trials.

*G. biloba* extract as dietary supplements does not require pre-marketing approval from regulatory agencies around the world. Furthermore, *G. biloba* extracts prepared by different manufacturers vary in the contents of bioactive compounds. Currently, there are no treatment recommendations of *G. biloba* extract for tinnitus. Existing evidence from RCTs remains inconclusive for the effectiveness of *G. biloba* extract.

### *G. biloba* in the treatment of tinnitus in mild to moderate dementia patients

Dementia is prevalent among the elderly and neurosensory symptoms, such as tinnitus and dizziness, are frequently reported in patients with dementia. However, dementia is often of mixed pathologies and defies treatment. *G. biloba* could offer a treatment option for dementia by reducing tinnitus, which is considered a sign of neurodegenerative disease and has been found to be independently associated with cognitive impairment (Jafari et al., 2019). Tinnitus is found to be a risk for development of Alzheimer’s disease (Chu et al., 2020). *G. biloba* is used for the treatment of early-stage Alzheimer’s disease and vascular dementia (Sierpina et al., 2003), and measures of tinnitus are now assessed in clinical trials of *G. biloba* extract in patients with dementia as earlier studies show that *G. biloba* extract could alleviate dementia-associated pathologies such as mitochondrial dysfunction and impaired hippocampal neurogenesis. In addition, tinnitus in patients with vascular dementia can represent an entity with a treatable cause given that *G. biloba* extract improves circulation (DeKosky et al., 2008).

*G. biloba* studies in the treatment of mild to moderate dementia are listed in Table 2. A double-blind RCT of 410 patients with mild to moderate dementia of vascular origin showed that treatment with *G. biloba* (Herrschaft et al., 2012) EGb 761,240 mg/day led to no improvement in the 11-Point Box Scale tinnitus score versus placebo. In a randomized, placebo-controlled, double-blind, parallel-group, multicenter trial, Schneider *et al.* demonstrated that *G. biloba* extract 120 mg or 240 mg for 26 weeks caused a -2.05 ± 2.09 reduction in the 11-Point Box Scale tinnitus score versus -.21 ± 1.69 with the placebo (Schneider et al., 2005). A recent meta-analysis (Spiegel et al., 2018) of five randomized placebo-controlled trials in 1972 patients with mild to moderate dementia showed a mean reduction in tinnitus severity in patients treated with *G. biloba* extract EGb 761, with a weighted mean difference of -1.06 (95% CI: 1.77, -.36) for tinnitus (*p* = .003), which corresponded to an improvement over placebo by 27%–40% of baseline severity in the individual studies.

**TABLE 2:** *G. biloba* in the treatment of mild to moderate dementia.

	Study design (trial registration)	Patients	Interventions	Outcomes
*G. biloba* Schneider et al. (2005)	Randomized, placebo-controlled, double-blind, parallel-group, multicenter trial	Mild to moderate dementia (*n* = 513)	Patients received *G. biloba* 120 mg or 240 mg, or placebo for 26 weeks	A-2.05 ± 2.09 reduction in 11-Point Box Scale tinnitus score vs. placebo -.21 ± 1.69
*G. biloba* EGb 761 Herrschaft et al. (2012)	Double-blind, placebo-controlled RCT	Mild to moderate dementia with neuropsychiatric features (*n* = 410)	Patients received a 240 mg once-daily formulation of G. biloba extract EGb 761 or placebo for 24 weeks	No difference in 11-Point Box Scale tinnitus score between patients receiving G. biloba extract EGb 761 and those receiving placebo
*G. biloba*EGb 761 (Napryeyenko and Borzenko, 2007)		Mild to moderate dementia with neuropsychiatric features (*n* = 400)	Patients received *G. biloba* EGb 761 or placebo for 22 weeks	A-2.11 ± 1.74 reduction in 11-Point Box Scale tinnitus score vs. placebo -.15 ± 1.01
*G. biloba* EGb 761 Ihl et al. (2011)		Mild to moderate dementia with neuropsychiatric features (*n* = 410)	Patients received 240 mg of *G. biloba* EGb 761 or placebo once daily for 24 weeks	A-1.11 ± 1.21 reduction in 11-Point Box Scale tinnitus score vs. placebo -.14 ± 1.92

Overall, these studies demonstrated that *G. biloba* extract benefited patients with mild to moderate dementia in terms of reducing the severity of tinnitus. However, measures of tinnitus were not assessed as a primary outcome in these trials of *G. biloba* extract for dementia and the results need to be interpreted with caution. In addition, depression and anxiety are prevalent in patients with tinnitus (Ziai et al., 2017). A mediation analysis showed that *G. biloba* extract EGb 761 directly accounted for 60% of the total effect of tinnitus severity reduction while amelioration of the symptoms of anxiety and depression and improvement in cognition contributed to 40% of the total effect (Brüggemann et al., 2021). The efficacy of *G. biloba* extract in reducing tinnitus severity in mild to moderate dementia patients remains to be investigated in vigorously conducted clinical trials with measures of tinnitus as the primary study end point.

## Other herbal medicines for the treatment of tinnitus

### Açaí

Herbal medicines other than *G. biloba* extract in the treatment of tinnitus are provided in Table 3. Açaí (Arecaceae; *Euterpe Oleracea* Martius), a fruit rich in α-tocopherols, fibers, lipids, mineral ions, and polyphenols, has been widely used for its anti-inflammatory and antioxidant properties (Benatrehina et al., 2018) It also contains flavonoids including catechin, chrysoeriol, anthocyanins and taxifolin and possesses high antioxidant capacity (de Liz et al., 2020; de Oliveira et al., 2021). Flavonoids (anthocyanins) in Açaí are suggested to be of potential activities for neurodegenerative diseases such as Parkinson’s disease (de Oliveira et al., 2019). In a double blind RCT, patients with chronic tinnitus received açai extract 100 mg or placebo (Oppitz et al., 2022). A significant reduction in THI score (-10.0 ± 6.4, *p* = .006) was observed in patients receiving açai extract 100 mg while no remarkable decrease in THI score was found in the placebo group (-8.8 ± 3.6, *p* = .093). A significant reduction in the Beck Anxiety Inventory (BAI) score was also observed in patients receiving açai extract 100 mg (*p* = .007). Though noted for its potent antioxidant capacity (de Liz et al., 2020; de Oliveira et al., 2021), Açai extract caused no significant changes in oxidative stress biomarkers. No other clinical evidence is currently available on the safety and efficacy of Açaí in patients with tinnitus. Additional trials are required to establish the efficacy of Açaí.

**TABLE 3:** Herbal Medicines other than *G. biloba* in the Treatment of Tinnitus.

Medicine herbs	Study design (trial registration)	Patients	Interventions	Outcomes
Açaí (*Euterpe Oleracea Martius*) Oppitz et al. (2022)	Double blind RCT (RBR-8z4mhq)	Chronic tinnitus (*n* = 30)	Patients received açai extract 100 mg or placebo	Mean difference from baseline THI -10.0 ± 6.4 and -8.8 ± 3.6
Korean red ginseng Kim et al. (2015)	Open-label RCT	Chronic tinnitus (n = 61)	Patients received Korean red ginseng 1,500 mg/day or 3,000 mg/day, or G. biloba extract 160 mg/day for 4 weeks	Mean difference from baseline THI 4.38 ± 2.31, 8.05 ± 2.33, and 4.05 ± 2.22
Gushen Pian Zhai et al. (2013)	Double blind RCT (Z20080046)	Non-hereditary acquired sensorineural deafness with associated tinnitus or simple tinnitus without hypoacusis (*n* = 120)	Patients received Gushen Pian 5 tablets (40 patients), Erlong Zuoci Pills (40 patients), or placebo (40 patients) for 4 weeks	Total effective rate, 89.2%, 74.3% and 30.8%; total relief rate, 59.5%, 57.1% and 5.1%
Bushen Huoxue Tongluo Zhang et al. (2022)	Assessor-blinded RCT	Chronic subjective tinnitus (*n* = 20)	Patients received Bushen Huoxue Tongluo granules and informative counseling, or informative counseling alone	The trial is ongoing. THI, tinnitus functional index, tinnitus sensation level, self-rated visual analogue scale on tinnitus loudness and annoyance, Pittsburgh Sleep Quality Index and adverse event

### Korean red ginseng

Korean red ginseng (Araliaceae; *Panax ginseng* C.A.Mey) has been used in folklore medicine for 2000 years and is currently being investigated for a variety of conditions including metabolic syndrome (Aminifard et al., 2021), inflammatory bowel disease (Kang et al., 2021) and diabetes (Liu et al., 2021). Studies have shown that Korean red ginseng has otoprotective activities including alleviation of cisplatin-induced ototoxicity *in vitro* and *in vivo* (Im et al., 2010), attenuation of noise-induced hearing loss (Mungan Durankaya et al., 2021) and amelioration of cochlear damage (Tian et al., 2013) and vestibular dysfunction (Tian et al., 2014). In an open-label RCT, patients with chronic tinnitus received Korean red ginseng 1,500 mg/day or 3,000 mg/day, or *G. biloba* extract 160 mg/day for 4 weeks. The authors found that only patients receiving Korean red ginseng 3,000 mg/day achieved a significant mean reduction in THI (-8.05 ± 2.33, *p* < .05). Notably, patients receiving Korean red ginseng 3,000 mg/day also showed significant improvement in role emotional and mental health assessed using short-form 36 (SF-36) (Kim et al., 2015). A recent meta-analysis of 36 RCTs were included with 2,761 participants showed that currently available agents including amitriptyline, acamprosate, and gabapentin, and intra-tympanic dexamethasone injection plus oral melatonin did not improve the quality of life despite demonstrable improvement in tinnitus severity and response rate of patients with tinnitus of no specific or treatable origin (Chen et al., 2021). Given that tinnitus severely impairs the quality of life of approximately 1%–2% of patients, it remains important to investigate the efficacy of Korean red ginseng and its effect on the quality of life of patients with tinnitus.

### Gushen Pian

Per traditional Chinese medicine theory, chronic subjective tinnitus is mainly caused by the insufficiency of essence to maintain normal kidney function, Qiu et al. (2015) stagnation and flow of blood in the meridian through the ear. Tonification of the essence of the kidney, improvement of blood flow, and dredging of the meridian passage around the ear could alleviate chronic tinnitus (Wang and Zheng, 2011). Gushen Pian, a herbal mixture consisting of *Drynaria fortunei*, *Danshen* (*Salvia miltiorrhiza Bunge* [Lamiaceae; Salviae miltiorrhizae radix et rhizoma]), licorice [Leguminosae/Fabacea; *Glycyrrhiza* glabra L.], and *Calcined Ci Shi*, has been reported to increase blood circulation, remove stasis, recuperate kidney, benefit essence of life and ventilate the ear (Zhai et al., 2013) and was found to be effective in the treatment of sensorineural deafness and hearing loss (Wang et al., 1996). In a double blind RCT (Z20080046) (Zhai et al., 2013), Gushen Pian displayed statistically significant therapeutic outcomes over placebo after 4 weeks of treatment with an overall effective rate of 89.2% versus 30.8% for placebo and an overall relief rate of 59.5% versus 5.1% for placebo for tinnitus. Currently, a pilot, assessor-blinded, randomized clinical trial (ChiCTR2100046632) of a traditional Chinese medicine formula, Bushen Huoxue Tongluo, is ongoing and will provide preliminary data on THI, self-rated VAS on tinnitus loudness and annoyance (Zhang et al., 2022). However, robust multicenter RCTs involving a large population size are still lacking.

## Limitations

The current review has several limitations. Despite abundant literature on herbal medicines for tinnitus, there are very few vigorously conducted clinical trials with measures of tinnitus as the primary study end point, which limits the effectiveness of the current analysis. In addition, *G. bibola* extract has not been standardized and different *G. bibola* extracts may be used in trials, making comparison across trials difficult. The conclusion of the current analysis may be constrained by the small sample size of the included trials. Furthermore, *G. biloba* compounds and their effects on tinnitus have not been investigated in randomized trials and further studies on the molecular mechanisms of action of *G. bibola* extracts should be conducted in the future.

## Conclusion

Tinnitus is a highly prevalent condition and becomes increasingly common with rising ages. Multiple mechanisms are implicated in the pathogenesis of tinnitus including maladaptive neuroplasticity (Shore et al., 2016), vascular dysfunction (Farri et al., 1998), oxidative stress (Celik and Koyuncu, 2018), and genetic disposition (Celik and Koyuncu, 2018; Wells et al., 2021; Xie et al., 2021). In addition, tinnitus is often found in patients with comorbidities such as depression and anxiety which may complicate treatment as well as assessment of effect of treatment in tinnitus patients. The heterogeneous nature of tinnitus suggests that combination treatment targeting diverse pathogenetic mechanisms and active management of comorbidities are required for effective management of tinnitus. *G. biloba* is the most widely investigated herbal medicine for tinnitus, but clinical trials have yielded conflicting results. Lack of *G* ***.*** *biloba* extract standardization, inadequate sample size, lack of optimization of treatment dose and duration, and poor study design are some of the issues hampering the study of *G. biloba* extract and other herbal medicines for tinnitus. As tinnitus is multifactorial, careful selection of subjects for a clinical trial is encouraged to minimize a variable population response and identify which patient subpopulation benefits from a particular treatment. Quality of life measures should also be incorporated into future studies given the impact of tinnitus on daily functioning of the patients.

Currently available evidence on herbal medicines for tinnitus is overall of insufficient quality and the conclusions from existing RCTs are contradictory. Nevertheless, the need is clear for effective pharmacotherapy for tinnitus, it is hoped that advances in basic science research on the mechanisms of tinnitus and further phytochemical and biological characterization of herbal medicines will eventually lead to a safe and effective pharmacological treatment for tinnitus.

## Abbreviations

AAO-HNSF, American Academy of Otolaryngology-Head and Neck Surgery; RCTs, randomized controlled trials; THI, Tinnitus Handicap Inventory; VAS, Visual Analogue Scale; TFI, Tinnitus Functional Index; TQ, Tinnitus Questionnaire.
